# Decoding microRNAs in autism spectrum disorder

**DOI:** 10.1016/j.omtn.2022.11.005

**Published:** 2022-11-07

**Authors:** Jinyu Li, Xiaohui Xu, Jiane Liu, Sudan Zhang, Xiaohua Tan, Zhiqiang Li, Jian Zhang, Zheng Wang

**Affiliations:** 1 Department of Genetics and Cell Biology, Basic Medical College, Qingdao University, Qingdao, Shandong 266071, China; 2 Department of Reproductive Medicine, the Affiliated Hospital of Qingdao University, Qingdao, Shandong 266000, China; 3 The Affiliated Hospital of Qingdao University & the Biomedical Sciences Institute of Qingdao University, Qingdao Branch of SJTU Bio-X Institutes, Qingdao University, Qingdao, Shandong 266003, China; 4 Department of Medical Genetics, School of Basic Medical Sciences, Southern Medical University, Guangzhou, Guangdong 510515, China

**Keywords:** MT: Non-coding RNAs, ASD, miRNA, disease modeling, therapeutic strategies, biomarker

## Abstract

Autism spectrum disorder (ASD)—a congenital mental disorder accompanied by social dysfunction and stereotyped behaviors—has attracted a great deal of attention worldwide. A combination of genetic and environmental factors may determine the pathogenesis of ASD. Recent research of multiple ASD models indicates that microRNAs (miRNAs) play a central role at the onset and progression of ASD by repressing the translation of key mRNAs in neural development and functions. As such, miRNAs show great potential to serve as biomarkers for ASD diagnosis or prognosis and therapeutic targets for the treatment of ASD. In this review, we discuss the regulatory mechanisms by which miRNAs influence ASD phenotypes through various *in vivo* and *in vitro* models, including necropsy specimens, animal models, cellular models, and, in particular, induced pluripotent stem cells derived from patients with ASD. We then discuss the potential of miRNA-based therapeutic strategies for ASD currently being evaluated in preclinical studies.

## Graphical abstract

**Figure:**
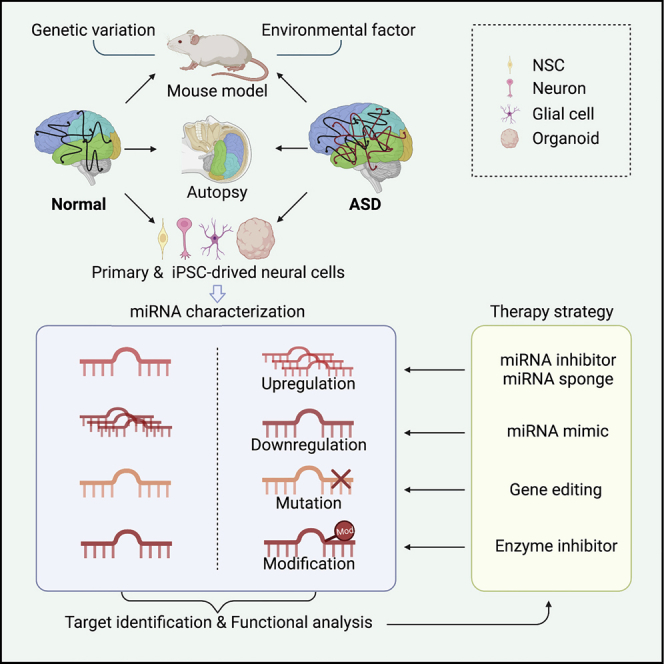

## Teaser

Various ASD models demonstrated dysregulation of key ASD-linked miRNAs and several mechanisms by which miRNAs influence ASD outcome. Therefore, miRNA-based therapy represents a promising avenue for the treatment onset and progression of ASD after further addressing major knowledge gaps and developing appropriate therapeutic approaches.

## Introduction

Autism spectrum disorder (ASD) is characterized by impaired social communication and restricted, ritualistic patterns of behavior accompanied by abnormalities in cognition, learning, memory, and sensory processing.^1^^,^^2^ It is worth noting that the prevalence of autism is rising at a frightening rate. Centers for Disease Control and Prevention estimated that, in the United States, ASD incidence rate was 1.85% as of 2016, an increase of 178% from 2000.^3^ More recent ASD data in Denmark and Japan also showed that the cumulative incidence rate would exceed 2.8% and 3.1%, respectively.^4^^,^^5^ Typically, symptoms of ASD appear early in life, especially between the ages of 2 and 5, and persist throughout life.^6^ The ratio of male incidence rates relative to female rates is 4:1.^7^ In addition, the symptoms can vary greatly, and there are no two ASD children who exhibit exactly the same symptoms. Such highly heterogeneous disease presentation poses great challenges in elucidating the pathogeny or in investigating the pathogenesis of ASD.^8^^,^^9^ Therefore, patients with ASD often have missed or late diagnoses, and currently there is no specific treatment in clinic.^10^

The etiology of ASD is usually described as a combination of genetic and environmental factors.^11^ In some studies of autistic families and twins, the genetic contribution can reach up to 90%, and the siblings of children with ASD have a higher risk of ASD.^12^ Genome-wide association studies, next-generation sequencing, and other technologies are widely used to screen genes linked to ASD. *MECP2*,^13^^,^^14^ *SHANK3*,^15^^,^^16^ and *FMR1*^17^ were reported as high-confidence ASD candidate genes, and dysregulation of *NRXN1* could decrease the neuronal activity in the brain of ASD patients.^18^ Of interest, microdeletion of 22q11.2,^19^^,^^20^ microdeletion or microduplication of 16p11.2,^21^ and microduplication of 16p13.11^22^ are also genetic links to susceptibility for ASD. As such, ASD has been initially considered a monogenic/polygenic disorder.^23^ However, growing evidence suggested that, in addition to genetics, environment is a more common cause of ASD. Infections, such as viruses and bacteria,^24^^,^^25^ stress,^26^ nutritional intake,^27^ alcohol exposure,^28^ and parental obesity^29^ were all recognized as risk factors of ASD. Accordingly, multiple *in vitro* and *in vivo* ASD models, including necropsy specimens, mice models, and cell models were developed to elucidate the underlying mechanisms in ASD and, in particular, to evaluate whether the association between genetic and environmental risk is a potential trigger of ASD phenotypes.

MicroRNAs (miRNAs) are a class of small single-stranded non-coding RNA containing approximately 22 nucleotides that repress the posttranscription of target genes.^30^ In the process of miRNA biosynthesis, DROSHA and DiGeorge syndrome critical region gene 8 (DGCR8; also known as Pasha) form a complex to cleave primary miRNA (pri-miRNA) into precursor miRNA (pre-miRNA).^31^ DICER, a protease belonging to the RNase III family, specifically recognizes and cuts double-stranded RNA into multiple small fragments of RNA, namely small interfering RNA (siRNA), which guides the RNA-induced silencing complex (RISC) to degrade the target mRNA.^32^ It is now clear that miRNAs mediate neural development, which is a complex process reviewed elsewhere.^33^^,^^34^^,^^35^ The overall function of the miRNA pathway in ASD has been checked in zebrafish,^36^ mouse,^37^ and humans^38^ by analyzing the phenotypes of *DGCR8* and Argonaute 1 (a component of RISC together with Argonaute 2) mutants, respectively, highlighting that miRNAs may act as a central player at onset and progression of ASD. In particular, miRNA expression levels and miRNA-mRNA regulatory networks as well as gene variants encoding miRNA (such as copy-number variations [CNVs] and single-nucleotide polymorphisms [SNPs]) have been widely studied in ASD. Especially in recent years, miRNA has been found to play a vital role in elucidating the interplay of genetics and the environment in ASD. In this review, we summarize recent advances of the emerging roles of miRNAs in controlling neural differentiation, synaptic plasticity, memory formation, inflammation, and dendritic development in ASD with particular emphasis on new mechanisms uncovered from various *in vitro* and *in vivo* models. Finally, we briefly discuss current efforts to therapeutically target specific ASD-associated miRNAs in preclinical studies.

## miRNAs in autopsy brain tissues of ASD cases

Autopsy brain tissues with high quality were considered to be a useful substrate to study neurological disorders and also represent the most direct model for exploring the neurobiological basis of ASD. Several important studies employed autopsy samples to examine differential miRNA expression in various brain regions of ASD patients.

### Cerebellar cortex

**Figure 1:**
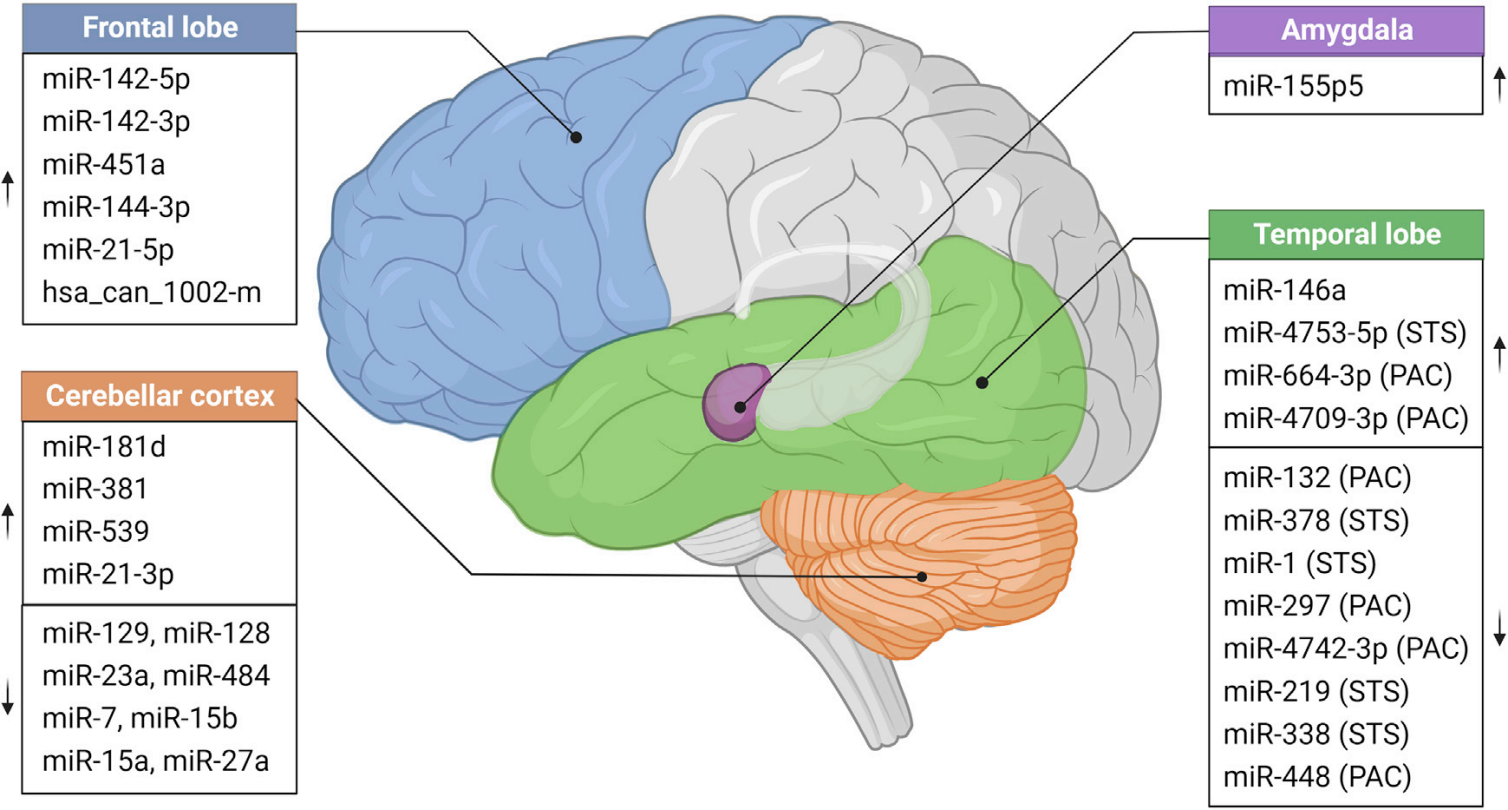
miRNAs misexpression in ASD patient autopsy brain Illustration of dysregulated miRNAs in different regions of ASD patient autopsy brain tissue, including cerebellar cortex (in orange), frontal cortex (in blue), temporal lobe (in green) (STS, superior temporal sulcus; PAC, primary auditory cortex), and amygdala (in purple). Upward arrow indicates up-regulated miRNAs, while downward arrow indicates down-regulated miRNAs.

In mammals, the cerebellum is in charge of individual emotions and movements.^39^ Previous studies have found abnormalities in the cerebellum of ASD patients through functional magnetic resonance imaging^40^ and neuroimaging techniques.^41^ Multiplex real-time PCR on ASD postmortem cerebellar cortex samples revealed a large cohort of abnormally expressed miRNAs, and some of them were predicted to repress key ASD-associated genes. Among these, miR-381, miR-181d, miR-23a, miR-128, miR-539, miR-27a, miR-328, miR-129, and miR-218 are predicted to target *NRXN1*, whereas miR-484, miR-7, miR-128, miR-328, miR-15a, miR-15b, and miR-27a potentially repress the translation of *SHANK3*.^42^ Of interest, miR-128, miR-27a, and miR-328 could target both *NRXN1* and *SHANK3*, suggesting that miRNAs may synergistically contribute to the ASD phenotype by modulating multiple key regulators of ASD (Figure 1). Moreover, some of these miRNAs have genetic variants present in the population. For example, clone-based comparative genomic hybridization analysis and SNP genotyping arrays identified MIR484 and MIR7 with CNVs, and SNPs rs895819 and rs11671784 were found in miR-27a. Nevertheless, whether these genetic variations within miRNAs could alter target genes’ expression and what that might entail have not been established. Likewise, by genome-wide miRNA expression profiling, a similar study showed that miR-21-3p was significantly upregulated in cerebellar cortex tissue samples, resulting in reduction of *DLGAP1*, which correlated with impaired postsynaptic density assembly and social ability.^43^^,^^44^

### Frontal cortex

Cognitive activity combined with neuroimaging studies have shown that the prefrontal cortex is the central regulator of social cognition, especially when analyzing people’s emotions.^45^ Small RNA sequencing of ASD autopsy brain tissue found that miR-142-5p, miR-142-3p, miR-451a, miR-21-5p, and miR-144-3p exhibit increased expression in Brodmann area 10, a subregion of the frontal cortex^46^^,^^47^ (Figure 1). Mechanistically, the promoter region of miR-142 was hypomethylated, as examined by Illumina 450K methylation array, suggesting epigenetic involvement in the imbalance of miRNA in the brain of ASD patients. In addition, luciferase assay revealed that miR-21-5p and miR-451a directly repress the expression of oxytocin receptor factors, regulators of complex social cognition, such as social exploration, anxiety, and aggression.^46^ In a more recent study, the primate-specific miRNA hsa_can_1002-m was diminished in ASD cerebral cortex, and this influenced a large cohort of genes associated with neural development and immune function due to their suppression of EGF and FGF signaling.^44^

### Temporal lobe

The temporal lobe underlies the function of social cognition, language, and psychological activities.^48^ In patients with ASD, the temporal lobe is impaired; positron emission tomography (PET) scans reveal that the blood perfusion is insufficient in this region.^49^ Expression analysis of the temporal lobe of ASD children revealed that miR-146a was the most up-regulated miRNA in the ASD brain in the early postnatal period compared with healthy controls (Figure 1). In these samples, high-confidence risk genes of ASD were downregulated, such as *SYT1* (patients with missense variants in *SYT1* showed blood perfusion insufficiency phenotypes in temporal lobe).^50^^,^^51^ Since then, several reports have revealed diverse mechanisms of miR-146a regulation in the temporal lobe. Ectopic expression of miR-146a predisposed human embryonic stem cell (line H9; WA09)-derived neural stem cell (NSC) differentiation to a neuronal-like fate, with neurite outgrowth, branching, and significant enrichment of ASD-linked genes, such as *FOXP2*, thereby resulting in aberrant neurodevelopment.^51^ Similarly, overexpression of miR-146a in mouse NSCs promotes neuronal differentiation as a result of cell-cycle arrest owing to Notch pathway inhibition.^52^ In support of this, complete miR-146a deficiency in mouse model disrupted the balance between apical radial glia and intermediate progenitors (two major types of NSCs co-exist in the neocortex of fetal mouse on embryonic day 14) and thus impaired neuronal differentiation and neurite branching, which resulted in learning and memory deficit.^53^ In addition to neurons, miR-146a induction in astrocytes increased glutamate uptake capacity and neuronal dendritic arborization,^54^ which, for the first time, provided the opportunity to link the miRNA regulation to high glutamatergic phenotype in ASD patients. More interestingly, the expression level of miR-146a decreased substantially after *Mecp2* deletion in the mouse ASD model,^55^ suggesting that either an increase or decrease of miR-146a has very likely been the driver of ASD phenotype.

To account for the intricate structure of the temporal lobe, more precise subregional analysis was conducted to delineate the miRNA expression between the superior temporal sulcus (STS; a temporal lobe structure that separates the superior temporal gyrus) and the primary auditory cortex (PAC; adjacent to STS, which functions to process information) in ASD patients and found higher similarity in miRNA expression between STS and PAC of ASD patients compared with normal individuals, with a complete loss of age-related alteration. For example, the expression of miR-132, which regulates the morphology of dendritic spines,^56^ decreased with age in the development of brain PAC, whereas this phenomenon was not observed in brains with ASD. On the other hand, the expression of miR-378 involved in regulation of learning and memory^57^ decreased with age in STS regions, which was not detectable in ASD brain.^58^ Further miRNA microarray analysis confirmed that this region associated dysregulation in the STS (up-regulation of miR-4753-5p and down-regulation of miR-1) and PAC (down-regulation of miR-297 and miR-4742-3p) of ASD patients^59^ (Figure 1). *In silico* analysis of data from the above studies found that specific miRNAs exhibit sex-based differences in expression—these abnormalities of miRNAs appeared more common in females than males, with STS regions accounting for the vast majority. For example, miR-338 and miR-219, which promote oligodendrocyte differentiation,^60^ as a result of inhibition of key transcriptional factors SOX6 and HES5,^60^^,^^61^ are significantly down-regulated in the STS region of female ASD patients but not males,^62^ whereas anxiety-related miR-488^63^ is down-regulated in the PAC of female ASD patients (Figure 1). These data may reflect the fact that ASD shows higher morbidity among males than females.

Within the temporal lobe, the amygdala is another important region responsible for emotional processing and storage,^64^^,^^65^ as well as for spatial and social exploration behaviors.^66^ Compared with non-ASD children, miR-155p5, a member of the miR-155 family, is largely increased in the amygdala, but not in dorsolateral prefrontal cortex, of ASD children (Figure 1). As a consequence, the expression of proinflammatory cytokine interleukin-6 (IL-6) was induced, thereby specifically promoting the glial lineage formation. This mechanism contributes to the inflammatory phenotype of ASD in the amygdala.^67^

## miRNAs in animal models of ASD

As a neurodevelopmental disorder, ASD normally emerges in early childhood. However, for pathology analysis, it is basically impossible to have access to the fetal postpartum brain tissue of ASD patients. In this regard, animal models recapitulating aspects of ASD would be applied as an appropriate choice. Mice models carrying known ASD mutations and induced by drugs or other substances are commonly used to explore the pathogenic mechanism of ASD *in vivo*.

### Mecp2 mutant mouse

The *MECP2* gene encodes a methylated DNA-binding protein, which can modulate the target genes by directly binding DNA methylated CpG islands, and then recruits other regulatory factors to fulfill repressive functions.^68^ Mutation of *MECP2* causes Rett syndrome (RTT), a devastating neurodevelopmental disorder with an autistic phenotype.^69^ By contrast, an increase of MECP2 expression as a result of *MECP2* gene duplication also leads to severe autism symptoms (known as MeCP2 duplication syndrome).^70^

**Figure 2:**
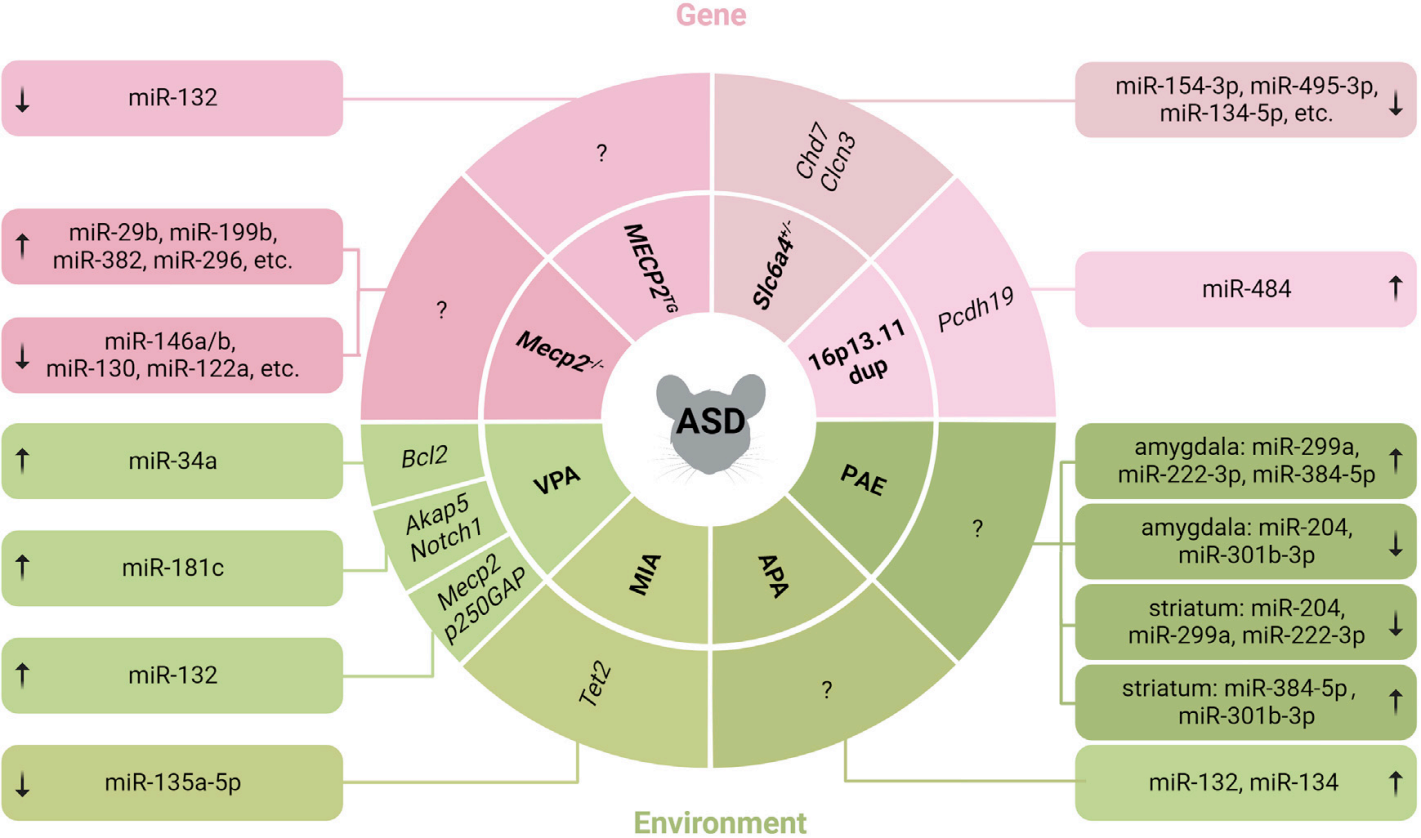
Dysregulated miRNAs in ASD animal models Genetic mutations (top pink ones) involve *Mecp2* mutation, *Slc6a4* heterozygous knockout, and 16p13.11 microduplication; environmental factors (bottom green ones) include poly(I:C), VPA, PAE, and APA. Both of them cause dysregulation of miRNAs and their targets, leading to the ASD phenotype in mice model. Upward arrow indicates up-regulated miRNAs, while downward arrow indicates down-regulated miRNAs. Question marks indicate miRNA target genes to be determined. *Mecp2*, methyl-CpG binding protein 2; *MECP2*^*TG*^, MECP2 transgenic mice; *Slc6a4*, solute carrier family 6 member 4; PAE, prenatal alcohol exposure; APA, advanced paternal age; MIA, maternal immune activation; VPA, valproic acid; *Chd7*, chromodomain helicase DNA-binding protein 7*;Clcn3*, chloride voltage-gated channel 3; *Pcdh19*, protocadherin 19; *Bcl2*, B cell lymphoma 2; *Akap5*, A-kinase anchoring protein 5; *Notch1*, Notch receptor 1; p250*GAP*, Rho GTPase-activating protein 32; *Tet2*, Tet methylcytosine dioxygenase 2; poly(I:C), polyinosinic:polycytidylic acid.

miRNA expression microarrays analysis of wild-type and *Mecp2*-knockout mouse brains revealed wide variations—down-regulated miRNAs were miR-146a, miR-146b, miR-342, miR-122a, miR-130, and miR-409, and up-regulated miRNAs included miR-29b, miR-199b, miR-382, miR-221, miR-296, miR-329, and miR-92^55^ (Figure 2). Of these, miR-146a, as well as miR-146b, have been the most extensively studied, the expression of which are completely lost in *Mecp2*-knockout mouse brains, in contrast to the marked induction in the temporal lobe that is discussed above.^51^ Indeed, ectopic expression of miR-146a or miR-146b in the mouse neuroblastoma Neuro-2a cell line resulted in the down-regulation of IL-1 receptor-related kinase 1 (IRAK1; an enzyme involved in proinflammatory immune responses),^55^ consistent with the marked *IRAK1* increases in patients with RTT with *MECP2* mutations.^71^ Surprisingly, both *IRAK1* and *MECP2* expression levels increased by more than 2-fold in lymphoblasts,^70^ probably due to the genomic region spanning of *MECP2* and *IRAK*s, indicating that *MECP2* may crosstalk *IRAK*s in the multilayer in a cell-type-dependent manner. However, in patients with *MECP2* duplication syndrome, whether miR-146a and miR-146b are repressed or not and what other miRNAs are involved in the *IRAK1* regulation are still unknown. Likewise, ectopic expression of miR-130a, a member of the miR-130 family that is also significantly decreased in *Mecp2* knockout mice^55^ led to inhibition of neurite growth and reduction of dendritic spine density and complexity in cortical neurons derived from rat embryos. These effects are attributed to the direct repression of *Mecp2*, thereby establishing a feedforward loop between miR-130 and loss of *Mecp2*.^72^

ASD patients always present in the clinic with either heightened or reduced pain sensitivity. This symptom was first explained in an *MECP2* replication syndrome transgenic (*MECP2*^*TG*^) mouse model with alleviation of acute pain, highlighting an analgesic action of MECP2. Intriguingly, MECP2 was defined as a key modulator in attenuating chronic pain as well^73^ (Figure 2). This fascinating synonym was also detected by a later study using *in vitro* cultured mouse cortical neurons.^74^ Thus, the *MECP2*-miRNA-mediated pain transduction reflects a new sensory mechanism in ASD.

### Slc6a4-knockout mouse

Previous studies revealed that alterations of *Slc6a4*, a serotonin transporter responsible for transmitting serotonin in synaptic space to presynaptic neurons, under prenatal stress are implicated in ASD phenotypes, such as emotional and behavioral abnormalities.^75^ Next-generation sequencing analysis of fetal brain of heterozygous *Slc6a4*-knockout mouse embryos with the maternal acute restraint stress at embryonic day 12.5 (E12.5) found a heavy increase of total methylation level genome wide accompanied by abnormal expression of several miRNAs. Among them, miR-1224-5p, miR-292a-5p, miR-760-3p, miR-331-3p, miR-874-3p, miR-134-5p, miR-154-3p, miR-495-3p, miR-376b-3p, and miR-299a-3p were down-regulated, whereas miR-21c, miR-135a-5p, and miR-16-5p were up-regulated.^76^ Intriguingly, all these miRNAs were predicted to target ASD-linked genes, such as *CHD7*^77^ and *CLCN3*,^78^ which are related to neuronal development and neuron adhesion, respectively (Figure 2).

### 16p13.11 transgenic mouse

Several lines of evidence indicated chromosome 16p13.11 microduplication as a risk factor implicated in several neurodevelopmental disorders, such as ASD,^79^ developmental delay, intellectual disabilities, and attention-deficit/hyperactivity disorder.^80^ miR-484, which is embedded in the 16p13.11 region, was speculated to contribute such neurocognitive deficit.^22^ To this end, transgenic mice carrying human 16p13.11 locus (16p13.11 dup) was generated, showing that induction of miR-484 expression promoted cortical neurogenesis by inhibiting protocadherin-19 (*Pcdh19*)^81^ (Figure 2). PCDH19 is a calcium-dependent cell-cell adhesion molecule that is mainly detected in the brain, and its mutants were found in ASD.^82^ Therefore, neurogenesis deficit owing to misexpression of the axis of miR-484/*Pcdh19* leads to the phenotypes of 16p13.11 microduplication syndrome.

### Maternal immune activation

Epidemiological^83^ and experimental^84^ studies show that maternal immune activation (MIA) triggered by infection during pregnancy is an important risk factor of ASD in offspring. Polyinosinic:polycytidylic acid (poly(I:C); a synthetic double-stranded RNA that mimics viral infection and activates an immune response) produces dose-dependent cytokine responses (IL-6, IL-12, and tumior necrosis factor α [TNF-α]) equivalent to responses observed in naturally occurring or opportunistic viral infections^85^^,^^86^ and, more importantly, induces ASD phenotypes, such as reduced social preference^87^ and stereotyped behaviors.^79^ miRNA microarray analysis of brain tissue of 3-week-old MIA fetuses induced by poly(I:C) identified a decent number of abnormally expressed miRNAs (8 up-regulated and 21 down-regulated miRNAs), accompanied by 758 differentially expressed mRNAs analyzed by RNA sequencing. Gene Ontology analysis showed that the up-regulated mRNAs were mainly enriched in methylcytosine dioxygenase activity. For example, *TET2*, a member of the *TET* family of enzymes mediating DNA demethylation, is largely up-regulated due to the down-regulation of miR-135a-5p^88^ (Figure 2). This discovery supports the notion that miRNA dysfunction mediates DNA methylation, thereby increasing susceptibility of ASD.^89^

### Valproic acid exposure

Valproic acid (VPA) is a short-chain fatty acid that is highly teratogenic as an antiepileptic drug.^90^ Exposure to VPA *in utero* increases the risk of cognitive impairment.^91^ In a 5-year prospective study, children of women exposed to VPA monotherapy had significantly lower intelligence quotient scores at age 3 than those exposed to other antiepileptic drugs.^92^ A VPA-induced ASD rat model of postnatal day 90 (P90) revealed the increase of miR-181c in the amygdala. Mechanistically, rat primary amygdala cells treated with sponge-miR-181c identified the downstream target genes, including *ApoE*, *S100b*, *Grasp*, *Akap5*, *Ngr1*, and *Notch1*, which function in dendritic growth and branching and spinal development.^93^ In a similar study, miR-34a, previously identified as a repressor of *Shank3* in mouse hippocampal neurons,^94^ was ranked the top up-regulated miRNA in the cerebellar cortex of VPA-exposed mice. Activation of miR-34a directly targeted *Bcl2* at E18 and P14,^95^ consistent with the loss of BCL2 levels in the parietal lobe of ASD patients,^96^ whereas CD1 mice exposed to VPA at E12.5 showed increased levels of miR-132 and decreased expression of target genes, including *Mecp2* and Rho GTPase-activating protein p250GAP, which is a key suppressor of axon branching^97^^,^^98^ (Figure 2). Unexpectedly, when the maternal mice were exposed to VPA at E14.5, the expression of miR-132 in embryonic brain did not alter,^99^ implying that the miRNA-responsive susceptibility of fetal brain to harmful substances depends on the stage of pregnancy a patient is in.

### Prenatal alcohol exposure

It is described that prenatal alcohol exposure (PAE) can impair neurodevelopment and cause ASD-like social disorders in offspring.^100^ After alcohol exposure at E12.5, the offspring of rats showed reduced social motivation, which could be reversed by social enrichment—co-feeding with the offspring of rats without alcohol exposure. The possible mechanism is driven by the reversal of miRNA and mRNA expression in the amygdala and striatum (located within subcortical basal ganglia of the forebrain and coordinates multiple aspects of cognition^101^). After social enrichment, the up-regulation of miR-299a, miR-384-5p, and miR-222-3p and the down-regulation of miR-204 and miR-301b-3p may alter the cell dividing by disruption of cell-cycle signaling pathways in the amygdala, whereas in the striatum, miR-204, miR-299a, and miR-222-3p were down-regulated, while miR-384-5p and miR-301b-3p were up-regulated, and the mRNA targeted by these miRNAs is mainly enriched in the cell death signaling pathway^102^ (Figure 2). This suggests that the above reversed miRNAs, when in conditions of social enrichment, may have therapeutic potential to alleviate social phenotypes in ASD.

### Advanced paternal age

Recent evidence suggests that paternal aging can promote the development of ASD through epigenetic modification—changes in DNA methylation profiles in sperm of aging males may increase the risk of ASD in offspring.^103^ Advanced paternal age (APA) can damage children’s neurocognition, which is manifested in the occurrence of children’s ASD.^104^^,^^105^ miR-134 and miR-132 in the hippocampus of offspring rats at APA were significantly increased^106^ (Figure 2). Mechanistically, miR-134 can promote the growth of dendrites in rodent hippocampal neurons,^107^ and miR-132 facilitates the maturation of dendrites in newborn neurons,^108^ which may contribute to impaired social behavior and increased repetitive and stereotypical behaviors.

## miRNAs in ASD patient-derived iPSCs

Though rodent models are attractive for studying neurodevelopment and related diseases for many reasons, it is difficult and sometimes impossible to represent a neuropsychiatric disorder. In addition, single-cell transcriptomics and other analyses of ASD characterized varying molecular dysregulations across the different cell types in patient brain samples.^109^^,^^110^ As such, several cellular ASD models were applied to determine cell-type-specific ASD-linked pathology. The common cell types used to study miRNA in ASD include mouse primary hippocampal cells, cortical cells, and neuroblastoma cell lines. However, these cells fail to resemble what they are *in vivo* after several passages in *in vitro* culture or neoplastic transformation. Recently, patient-derived induced pluripotent stem cells (iPSCs) with specific pluripotency factors, carrying same genetic background and retaining partial epigenetic memory of their tissue of origin, could mimic all cell types in the brain by the advantage of their pluripotency potential. Therefore, these cells are displaying great potential to overcome obstacles that mouse models face and can give rise to patient-specific materials that will enable the dissection of the molecular mechanism underlying ASD in one model.^111^

### iPSCs from patients with 22q11.2 deletion or 16p11.2 microdeletion

**Figure 3:**
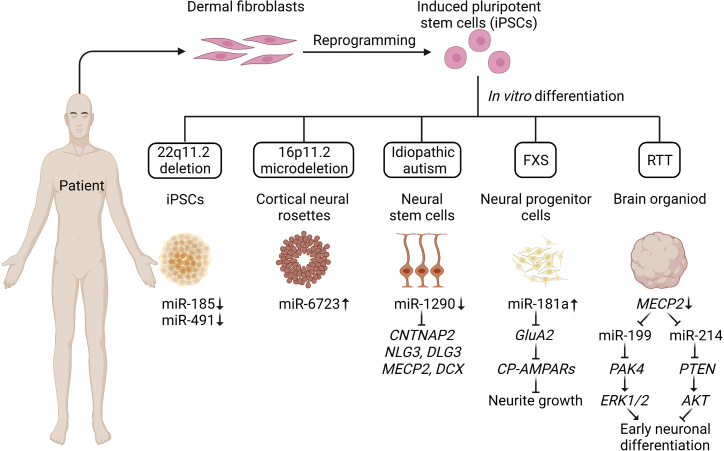
Mechanisms of deregulated expression of miRNAs in patient-specific iPSC disease modeling Somatic cells from ASD patients or healthy individuals can be reprogrammed to iPSCs and subsequently differentiated into cortical neural rosette, neural stem cell, or neural progenitor cell in two-dimensional (2D) culture or brain organoid in 3D culture in a stepwise process. These patient-derived cellular models allow for the identification of ASD-individual-related miRNAs and their targets, which potentially can be served as suitable candidates of personalized medicine. FXS, fragile X syndrome; RTT, Rett syndrome. *CNTNAP2*, contactin-associated protein 2; *NLG3*, neuroligin 3; *DLG3*, discs large MAGUK scaffold protein 3; *DCX*, doublecortin; *GluA2*, glutamate ionotropic receptor AMPA type subunit 2; *PAK4*, P21 (RAC1) activated kinase 4; *PTEN*, phosphatase and tensin homolog; *ERK1/2*, mitogen-activated protein kinase 1; *AKT*, AKT serine/threonine kinase; *CP-AMPARs*, calcium-permeable α-amino-3-hydroxy-5-methyl-4-isoxazole-propionic acid receptor.

22q11.2 deletion syndrome (22q11DS; also known as DiGeorge syndrome) is a frequent (1 in 4,000 people) genetic deletion in humans with variable neuropsychiatric diseases.^112^ This microdeletion accounts for 30%–40% of the risk of ASD,^113^ and it was reported that 15%–50% of patients with DiGeorge syndrome are on the ASD spectrum.^114^ Among the possible candidate genes in the 22q11.2 region are *DGCR8*^115^ and the *MIR185* gene,^116^ which encodes miRNA-185, a well-studied miRNA in ASD. Whole-transcriptome miRNA sequencing on human neurons differentiated from iPSCs that were derived from healthy individuals and patients with 22q11.2 microdeletion revealed two significantly down-regulated miRNAs—miR-185 and miR-491—along with another 25 miRNAs that were found to also be decreased in the hippocampus and prefrontal cortex of mice lacking *Dgcr8*^37^^,^^117^ (Figure 3). miR-185 has been found to be a repressor of *RhoA*, *Cdc42*, *Serca2*, and other schizophrenia-linked genes, dampening dendritic spine density in the hippocampus, suggesting it may participate in neuronal development and synaptogenesis exclusively.^118^ Notwithstanding the currently known regulatory function of miR-185, how the dysregulation of miR-491 influences neuronal function and triggers ASD remains unclear. miR-491 is very likely to influence synaptic plasticity in the amygdala owing to impulsivity and co-morbid traits given that amygdala size was found to be aberrant in patients with 22q11.2 microdeletion.^119^ Overall, the down-regulation of miR-185 and DGCR8-mediated miRNA biogenesis dysregulation contribute to the pathogenesis of 22q11DS.

It is reported that CNVs of the human chromosomal region 16p11.2 are another common genetic variant in ASD.^120^ After reprogramming skin fibroblasts from 16p11.2 microdeletion patients into iPSCs, cortical neural rosettes were successfully generated by direct differentiation. The expression of miR-6723 in the transcriptome of cortical neural rosettes was highly up-regulated^121^ (Figure 3). Further analysis confirmed that miR-6723 is specifically expressed in neurons compared with other cell types in the brain,^122^ although the mechanisms of action of miR-6723 at the onset of ASD are unclear.

### iPSCs from idiopathic autism

Idiopathic autism is diagnosed in subjects who have no known genetic cause of ASD and no related disorder on the spectrum.^123^^,^^124^ These patients’ iPSC-derived neuronal progenitors and organoids exhibited accelerated cell-cycle phenotype, resulting in loss of neuronal marker expression, accompanied by down-regulation of miR-1290.^125^^,^^126^ Indeed, synthetic miR-1290 treatment can inhibit cell-cycle abnormality.^127^ In addition, several other studies confirmed that miR-1290 targets several ASD-related genes such as *MECP2*, *DCX*, *DLG3*, *NLG3*, and *CNTNAP2*^127^^,^^128^^,^^129^ (Figure 3).

### iPSCs from RTT or fragile X syndrome

Isogenic iPSC-derived neurons from human patients with RTT showed induction of miR-199 and miR-214, which are direct targets of *MECP2*, phenocopying the cerebral organoids from iPSC in healthy controls treated with *MECP2* short hairpin RNA. It is well documented that miR-199 can inhibit the expression of PAK4 and ERK1/2, thereby impairing early neural differentiation, whereas miR-214 can repress the expression of PTEN and promote the expression of AKT, and then inhibit early neural differentiation^13^ (Figure 3).

Fragile X syndrome (FXS) is another leading cause of ASD. The genetic mutation responsible for FXS is *FMR1*. FMRP, encoded by the *FMR1* gene, is required for proper brain development.^130^ NSCs differentiated from patient-derived iPSCs carrying an *FMR1* mutation exhibited increased expression of miR-181a, a high homolog of miR-181c and miR-181d, which reduced the expression of GluA2, a subunit of glutamate analog receptors (AMPARs), leading to the increase of Ca2^+^-permeable AMPARs (CP-AMPARs) and consequent reduction of neurite growth^131^ (Figure 3).

## miRNA in ASD therapy

With a better understanding of the critical miRNA regulatory network controlling the ASD development, targeting miRNA in ASD possesses promising potential as a therapeutic intervention. miRNA-based therapeutics involve miRNA mimics and inhibitors (miRNA mimics are used to supplement specific miRNAs, whereas miRNA inhibitors can silence target miRNA expression, a strategy similar to past innovations in RNAi therapeutics^132^). In 2016, the US Food and Drug Administration (FDA) approved nusinersen, which is an antisense oligonucleotide, the first drug to treat spinal muscular atrophy. In 2018, the FDA came through with a breakthrough drug—patisiran, which is a small interfering ribonucleic acid, to treat polyneuropathy in patients by specific inhibition of hepatic synthesis of transthyretin. It is thus not surprising that, currently, there are several miRNA-based therapeutic clinical trials for treatment of various severe diseases that are reviewed elsewhere.^133^^,^^134^^,^^135^

To date, in the ASD field, there is no miRNA-based drug that has entered into clinical studies or been approved by the FDA yet.^136^ However, several preclinical research studies demonstrated the efficacy and potential with the rapid development of ASD disease models. For example, a miR-1290 mimic can rescue the ASD phenotype in NSCs derived from idiopathic ASD patients.^127^ More recently, in a mouse model carrying two human *MECP2* alleles and no mouse endogenous allele, intracerebroventricular injection of human-specific *MECP2*-antisense oligonucleotide was shown to specifically hybridize with the *MECP2* transcripts and repress their translation to normal levels, rescuing the anomaly of exploratory behavior and learning ability,^137^ a striking finding when considering that too much reduction of MECP2 would also cause RTT.^137^

As discussed above, various brain regions and cell types are implicated in ASD onset and progression. Single-cell RNA sequencing of cortical regions of autopsy samples showed largely differentially expressed genes in the neurons of the upper 2–3 layers of the cerebral cortex and microglia compared with the control group,^109^ as well as neuroepithelial cells,^138^ oligodendrocytes, and astrocytes.^139^ So, how to regenerate the new cells to fulfill the function becomes an intriguing question to both researchers and clinicians. Direct generation of neurons from other cell types has been demonstrated by taking advantage of neuron specific transcription factors^140^ or small chemical molecules.^141^ However, the conversion efficiency by these approaches is extremely low, thereby limiting the use in clinic. miR-9/9^∗^ and miR-124, specifically expressed in brain tissue, have been confirmed to be potent reprogramming factors that induce a neuronal state by replacing the conventional transcriptional factors cocktail. Mechanistically, miR-9/9^∗^ and miR-124 may simultaneously reinforce the targeting of neuronal-specific transcription factors through a complex epigenetic regulation process.^142^ As these two miRNAs target multiple genes permissive for establishing the neuronal environment, and miRNA mimics and inhibitors can be delivered into cells by exosomes, conversion of other cell types into clinically relevant subtypes of neuronal cells may give rise to future avenues for miRNA-based therapeutics. For example, miR-9/9^∗^ and miR-124 can modulate the conversion of adult fibroblasts into mature neurons *in vitro*,^143^ and miR-365 mediates astrocyte reprogramming into functional neuron *in vivo*.^144^

## Conclusion and future perspectives

**Figure 4:**
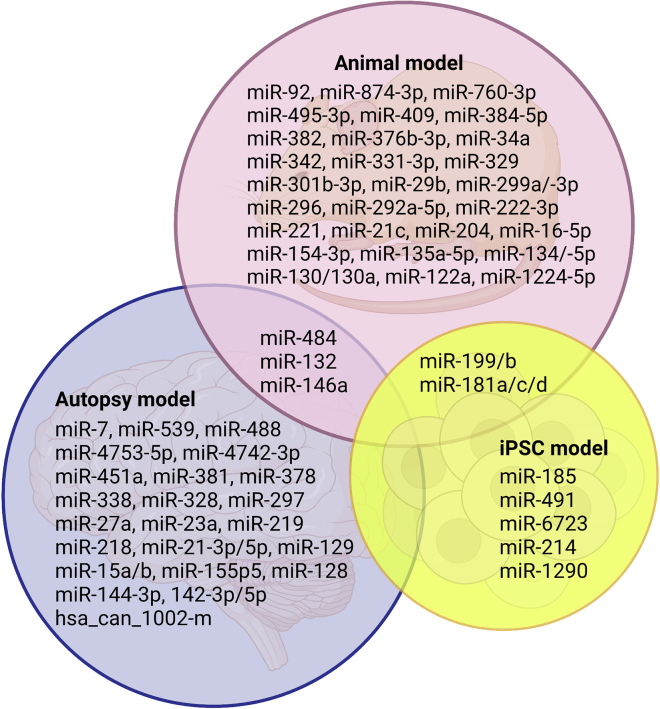
Common miRNAs associated with ASD in autopsy, animal, and iPSC models Venn diagram shows the overlap of misexpressed miRNA in autopsy (30 miRNAs, blue), animal (37 miRNAs, pink), and iPSC models (7 miRNAs, yellow).

Recently, the ASD and miRNA fields have converged with the dissection of ASD-specific miRNA, which is highlighted to link the genetic and environmental factors in brain functions. Understanding these miRNA dynamics in different regions of the brain is central to the understanding normal physiology as well as the pathophysiology of ASD, and addressing this important knowledge gap is the current challenge in the ASD field. With various ASD disease models, common and specific miRNAs associated with certain ASD settings would pave the way for novel diagnostic and therapeutic treatment of ASD (Figure 4).

Although several genes conferring susceptibility to ASD have been characterized, the identification of novel ASD-linked mutations remains an open question in the ASD field. Several categories of miRNA-associated mutations have been investigated heavily. (1) Mutations in genes encoding miRNA: whole-exome sequencing of lymphocytes or saliva extracted from ASD patients found SNPs within *MIR133B*/*MIR206*, leading to the regulation of *MET* gene,^145^ which subsequently hindered synaptic maturation.^146^ By analyzing CNV loci of miRNAs in the autism database, several CNV-miRNA were identified, including *MIR590*, *MIR944*,^147^ *MIR3618*, and *MIR1306*.^148^ (2) Mutations in sequences associated with miRNA biogenesis: mutations occurring in pre-miRNAs and neighboring regions can influence miRNA processing. *MIR934*-T/G transversion in ASD patients changes the cleavage site of DROSHA or DICER, which may alter the preference of RISC binding miRNA.^149^ (3) Mutations in the 3′ UTR region of miRNA target gene: SNPs present within 3′ UTRs of mRNAs perturbing miRNA-mediated gene regulation may cause the abnormal expression of autism-related genes, leading to the disease susceptibility or pathogenesis of at least some ASD patients.^150^ These observations highlight the understanding and assessment of these mutation susceptibilities, both genetically and environmentally, which allow for the development of therapies, in a more precise way, aimed at ASD prevention and treatment.

Notwithstanding the large amount miRNA expression data generated in ASD, a thorough knowledge of how miRNAs are differentially expressed temporally and spatially throughout ASD occurrence is demanding. Temporally, mice exposed to VPA on E12.5 could cause the increase of miR-132, and these phenomena were not detected when exposed on E14.5,^99^ which indirectly reflected the dynamics of miRNA expression. The solution for this would be to scan the expression of miRNAs at multiple time points during ASD onset and progression. Spatially, in the rat model of social enrichment after alcohol exposure, the dysregulated miRNAs in the amygdala and striatum are opposite for miR-299a, miR-301b-3p, and miR-222-3p.^151^ Recently, advances in spatial sequencing opened up the capability to molecularly characterize single cells within specific tissues by generating spatial transcriptomic data.^152^ Such data may allow us to decipher the region-specific dysregulation of miRNA and pave a path toward region- and cell-type-specific therapeutics.

Although miRNAs exhibit great potential in the treatment of diseases, their delivery, targeting, toxicity, and the *in vivo* stability are still great challenges for miRNA-based therapeutics.^132^ To summarize, understanding the miRNA regulation of brain development and maintenance is currently one of the biggest challenges in neuroscience research and the clinical treatment of neurological disease, such as ASD.
